# Electrophysiology of Single and Aggregate Cx43 Hemichannels

**DOI:** 10.1371/journal.pone.0047775

**Published:** 2012-10-24

**Authors:** Cole Brokamp, Jacob Todd, Carlo Montemagno, David Wendell

**Affiliations:** School of Energy, Environmental, Biological and Medical Engineering, Engineering Research Center, University of Cincinnati, Cincinnati, Ohio, United States of America; Temple University, United States of America

## Abstract

Connexin43 (Cx43) is the most ubiquitous gap junction protein in the human body and is essential for cell-to-cell communication in a variety of organs and organ systems. As a result, Cx43 is responsible for mediating both electrical and chemical signals, passing dissolved solutes and small signaling molecules between cells in a coordinated fashion. Here, we explore the electrophysiological properties of hemichannels formed from Cx43 and Cx43 fused to eGFP (Cx43eGFP) and their interactions in a planar lipid membrane (BLM). Unlike *in vivo* patch clamp experiments, Cx43 was purified and isolated from other membrane constituents allowing elucidation of individual protein responses to various electrical and chemical stimuli. Using this system, we examined hemichannel electrophysiology and the roles of several well-known gap junction blockers, namely: lanthanum, heptanol, carbenoxalone and lindane. We also observed a critical number of hemichannels required for an accelerated conductance increase, an emergent electrical signature indicative of plaque formation.

## Introduction

Cx43 has an essential role in cellular communication in a variety of tissues [1], [2]. Gap junction communication between two connexons is limited to molecules less than 1 kD, and single, unopposed hemichannels have been linked to a number of cellular processes including calcium waves, release of NAD^+^ and ATP, neuronal signaling, and the activation of several kinase cascades [3], [4].

Perhaps one of the most important functional aspects of gap junctions is the way the hexamer senses voltage and responds to it. Each hemichannel of a gap junction has its own voltage sensor, and several mutagenesis studies have shown that this voltage sensing is the cumulative effect of hemichannel subunit interactions [5]–[9]. Furthermore, it is how these interactions affect the partnering hemichannel which ultimately determines conductance and voltage gating properties of the gap junction. Therefore, it is the emergent electrical properties of assembled elemental connexin which determine the gap junction’s voltage sensitivity. There is considerable variety in the intercellular communication requirements for various organs, thus it is not surprising that there is a large diversity in connexin molecular weight and subunit assembly [10].

Given this complexity, we have sought to elucidate the role of purified individual homotypic hemichannels using isolated electrical and chemical studies. To date, only three investigations (Cx26, Cx32 and most recently Cx43), have been reported in planar bilayers, with the former two largely limited to electrical properties with varying degrees of success [11]–[13]. Here we explore both individual and cumulative Cx43 hemichannel conductance as well as the in-vitro effects of phosphorylation and several well-known connexin blockers.

## Materials and Methods

### Purification and Proteoliposome Formation

Cx43 was over-expressed using Sf9 cells (Life Technologies) and the InsectDirect system from Novagen (Life Technologies). Cells were grown at 28°C in a serum free media for 2 days after pIEX4 plasmid transfection carrying either Cx43, Cx43eGFP or Cx43_S368A DNA, all designed with enterokinase (EK) protease cleavage sites linking a c-terminal 6× his-tag. Rat Cx43 and Cx43eGFP cDNA were both gifts provided by Dr. Scott John (UCLA). The Cx43_S368A mutant was created by point mutation of the Cx43 pIEX4 construct using the Stratagene Lightning Mutagenesis kit as instructed. Finally all plasmids were verified by DNA sequencing between the IE promoter and terminator. Cells were grown in 75 cm^2^ flasks and harvested by centrifugation at 600 g’s. Purification was carried out as previously described [14]. Briefly, cells were lysed using a dounce homogenizer and the membranes were isolated by alkali extraction and centrifugation at 70,000 g’s for 45 minutes. Membranes were solubilized with 1% decylmaltoside and insoluble material was removed by centrifugation at 100,000 g’s for 1 hour. The supernatant was applied to a nickel-NTA column (GE Healthcare) washed and eluted with 20 mM and 500 mM imidazole, respectively. The c-terminal 6× his-tags were removed via digestion by EK using the Tag•off™ rEK Cleavage/Capture Kit (Life Technologies). The elution was further purified using a Sephacryl S300HR (GE Healthcare). The purified protein was analyzed by western blotting using a C-terminal specific Cx43 antibody (Sigma-Aldrich) (see Figure S1B). Proteoliposomes were formed in 1 M KCl, 5 mM MES, pH 5.7. 1,2-diphytanoyl-*sn*-glycero-3-phosphocholine (DPhPC) lipid (Avanti Polar Lipids) solubilized with *n*-octyl-ß-D-glucoside and added in a 100∶1 (w/w) ratio to protein. Formation of proteoliposomes was caused by an overnight removal of the detergent at 4 degrees Celsius by dialysis in the presence of biobeads (Biorad). A mock purification of Cx43 was also carried out as a control which involved the growth and harvesting of Sf9 cells devoid of the pIEX4-Cx43 plasmid. The output from the mock purification was then used in the same proteoliposome preparation and BLM fusion procedures (see Figure S1C). Proteoliposome size was normalized by extrusion through a 100 nm polycarbonate membrane to provide uniform vesicle fusion and protein insertion properties. When exposing dithiothreitol (DTT) and calf intestinal phosphatase (CIP, Sigma-Aldrich) to proteovesicles prior to injection, compounds were included before extrusion to ensure uniform exposure and distribution.

### Electrophysiology

A 500 µm diameter aperture in a Teflon partition separating two aqueous compartments was pre-painted twice with 0.5 uL of painting solution (0.5% DPhPC in decane) on both sides and allowed to dry for about 45 minutes. Both chambers, separated by the horizontal partition, were then filled with 500 mM KCl, 2.5 mM MES, pH 5.8. The painting solution was used to paint a membrane across the hole using a bubble at the tip of a micropipette. The ionic current through the membrane was measured with Ag/AgCl electrodes by using a patch-clamp amplifier (Axopatch 200B, Molecular Devices). The recordings were digitized with Clampex 9.2 software (Molecular Devices) at 5 kHz and filtered with a 1 kHz low pass Bessel filter. The membrane capacitance was monitored using the integration of a ramping voltage waveform until the appropriate single bilayer thickness was achieved, typically between 4–5 nm (see Text S1). Next, proteoliposomes were added to the chamber and allowed to fuse with the membrane. The experiment remained physically and electrically isolated by placing the headstage in a Faraday cage on top of a vibration isolation platform. Compounds such as NaOH and various blockers were dissolved in the chamber buffer and were added directly to the chamber using a pipette.

### Monitoring Vesicle Fusion Rate

Liposomes were generated using the same detergent removal protocol above except for the addition of 0.5 M ATP. After the liposomes were added to the top chamber, 250 µL of buffer from the bottom chamber was removed and replaced with chamber buffer every minute. A luciferase bioluminescence assay (Sigma-Aldrich) was used to determine the amount of ATP in each sample based a standard curve generated using known amounts of ATP.

## Results

### Activity in Planar Bilayers

**Figure 1:**
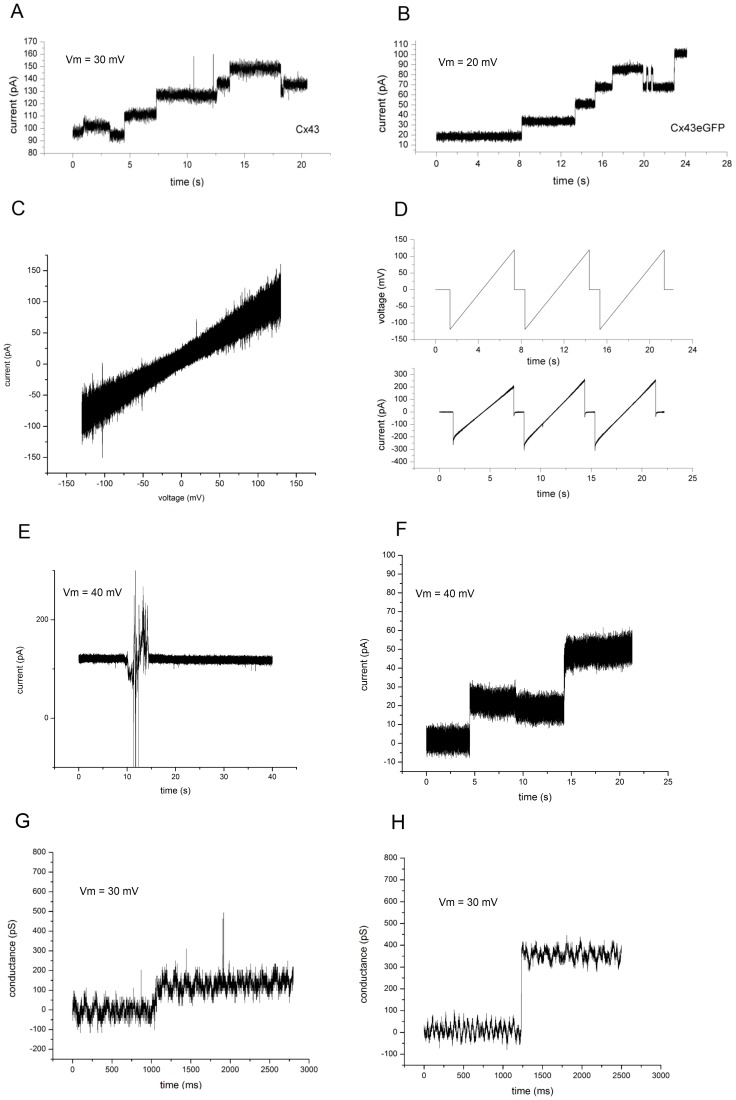
Cx43 and Cx43eGFP hemichannel conductance in a lipid bilayer. (A) Cx43 and (B) Cx43eGFP channels opening in a stepwise manner and exhibiting full and subconductive states. (C) Scan of Cx43 from −130 mV to +130 mV. (D) Action potential waveform that induced hemichannel openings in previous patch clamp studies [1], [2] did not cause increases in current when applied to Cx43 hemichannels. (E) When MES was added to a final concentration of 10 mM to the chamber buffer at pH 5.7, it did not affect the conductance of Cx43 hemichannels. (F) The activity of open Cx43 hemichannels inserting into the bilayer was not affected by using a chamber buffer containing 10 mM MES. (G) Cx43eGFP channel opening to a subconductive state of 35% of the full conductance. This transition lasts about 150 ms. (H) Two opposed Cx43eGFP hemichannels exhibiting a conductance of 50% of the full conductance of one hemichannel, as predicted by the series arrangement. This transition lasts less than 5 ms.

Exceptionally pure Cx43 protein, as observed in the combination of SDS Page and Cx43-specific western blotting, was prepared using insect cell over-expression and his-tag affinity chromatography as described previously [14]. This protein was then incorporated into lipid vesicles for fusion with a planar lipid bilayer, since purified protein solutions failed to insert Cx43 hemichannels spontaneously. Artificial bilayers were created with a gigaohm seal before proteoliposomes were added to the chamber. Proteoliposome fusion produced sharp, homogenous stepwise increases (Figure 1A and 1B) in conductance congruent with open channel insertions, suggesting incorporation of Cx43 and Cx43eGFP proteins. In contrast, liposomes prepared from a mock infection/protein preparation were also added to the artificial bilayer showing no changes in conductance (see Figure S1C). Channel current was measured at various holding potentials as well as at ramping voltages from −100 to +100 mV (Figure 1C) and a voltage waveform previously shown to evoke significant current increases at large positive bias for patch-clamped HeLa cells expressing Cx43 [15], [16]. Conductance was observed throughout the tested voltage range, and as a result of extrusion process, protein orientation within the vesicle membrane was randomized, making it difficult to assign specific orientations after fusion with the planar bilayer. The conductive states of hemichannels are dynamically regulated by pH, phosphorylation, extracellular calcium concentration, and voltage within the cell [17]. Consistent with the methodology for the two previous BLM hemichannel studies [11], [12], we found that keeping the BLM chamber buffer at pH 5.7 greatly increased the probability of open hemichannel insertion into the bilayer. After open hemichannels had inserted into the bilayer at pH 5.7, the chamber buffer was increased to pH 7.0 using a 500 mM NaOH solution causing an immediate loss of all hemichannel conductances. This is similar to previous studies in which a negative change in pH relative to the cytoplasmic face of the hemichannel caused channel closure [18]. Conversely, the addition of 500 mM HCl to only one side of the chamber in initially neutral buffer conditions (pH 7.0) caused hemichannel conductances to arise in previously inactive membranes. Our results echo the decade old hypothesis that the pH gradient across the pore, or “net balance of charge” may play a more critical role in pH based channel gating than the chamber buffer as a whole [19]. Also, the pH must be considered in conjunction with the aqueous buffering agent. Previous studies have shown that protonated sulfonates induce conformational changes in connexin hemichannels [18], completely blocking the channel [20]. For example, 10 mM MES directly and reversibly inhibits Cx26 hemichannels, but the pH-sulfonate-hemichannel relationship appears to be isoform specific, with MES and several other protonated sulfonates having no effect on Cx32 [20]. This same study showed connexin channel activity over a range of pH values from 5–8 using MES [20]. Recently, Tuarine was shown to block Cx43 hemichannels in a planar bilayer at pH 7 [13]. However, aminosulfonate channel blocking is sensitive to protanation state, and the pKa of Tuarine is 8.0, while MES is only weakly protonated at pH 5.8 [20]. When MES (10 mM final concentration) was added to a bilayer containing open Cx43 hemichannels, it failed to reduce the conductance (Figure 1E). Furthermore, using a chamber buffer with 10 mM MES did not reduce the activity of open hemichannels in the bilayer (Figure 1F).

**Figure 2:**
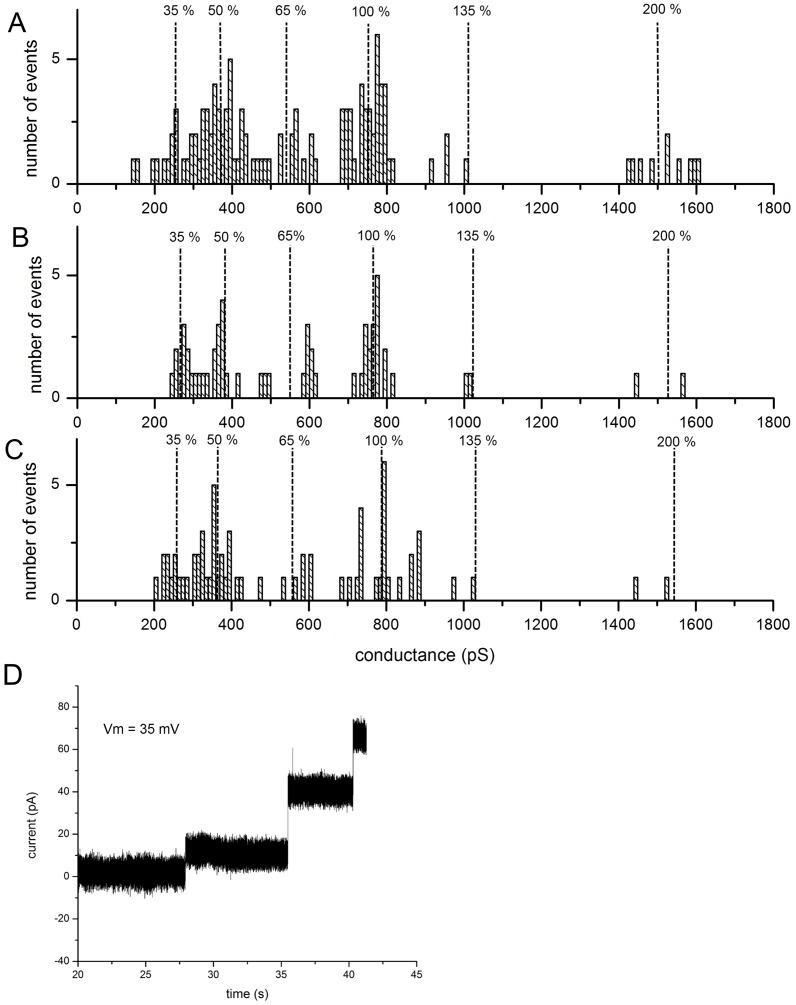
Connexin conductive states by frequency. The conductance changes observed from (A) Cx43 (B) Cx43eGFP and (C) Cx43_S368A channel openings are presented by frequency. The percentages of the respective single hemichannels are also plotted for comparison. The substate is 35% and the series conductance is 50%. (D) Vesicles pretreated with DTT showed the full and 35% subconductive states but no insertions at the 50% conductive state.

The average conductance of a single Cx43 hemichannel was found to be 753±31 pS (*n* = 30) for the 500 mM KCl buffer. Also in single hemichannel form, Cx43eGFP exhibited an average conductance of 783±53 pS (*n* = 30). Table S1 illustrates that our results compare well with a previous study in which the conductance of single Cx43 and Cx43eGFP hemichannels were recorded in HeLa cells using a patch clamp [15]. Although these measurements were carried out *in vivo,* an empirical conductance ratio between the buffers enabled a scaled comparison. While the full conductive states are similar to those found in the cellular investigations, one significant difference was the observation of subconductive states for both Cx43 and Cx43eGFP (Figure 1A, 1B and 1G). An example trace in Figure 1E shows a Cx43eGFP channel inserting while in a subconductive state of 35% of the open conductance. This corresponds very closely with the subconductive state found for Cx43 (35%) in patch-clamping studies [15]. A third conductance state was also observed for both Cx43 and Cx43eGFP equal to 50% of the full conductance of a single hemichannel (Figure 1H). This is thought to arise from a hemichannel inserting into the bilayer while docked with an opposed hemichannel since a series arrangement predicts twice the resistance for a full gap junction. Treating the proteoliposomes with dithiothreitol (DTT) to reduce disulfide bonds between external loops of individual hemichannels [21], and thus destabilize full gap junctions [21], abolished the 50% subconductive state (Figure 2D) but not the 35% state. Since connexin external loops are responsible for the connexon-connexon hydrogen bonding, destabilization of the loops would theoretically lead to destabilization of the paired hemichannels through the dislocation of interdigitated loops. The frequency of the conductance change events for all Cx43 types are compiled in Figure 2.

### Conductance Changes for Open Cx43eGFP Hemichannel Populations

In a mechanism similar to connexon biogenesis in cells, our connexons were delivered to the artificial bilayer in 100 nm vesicles [22]. The vesicles were sized by extrusion and the lipid to protein ratio was chosen to facilitate one hemichannel per vesicle [14]. After vesicles were added, discrete increases in conductance were observed consistent with the fusion of a single Cx43eGFP hemichannel from a single vesicle. Because of the variance in open and closed states of the hemichannels, it is also likely that hemichannels in a closed state inserted into the membrane without exhibiting any conductance. Once in the membrane, the hemichannels are free to laterally diffuse and interact with other hemichannels in the membrane.

**Figure 3:**
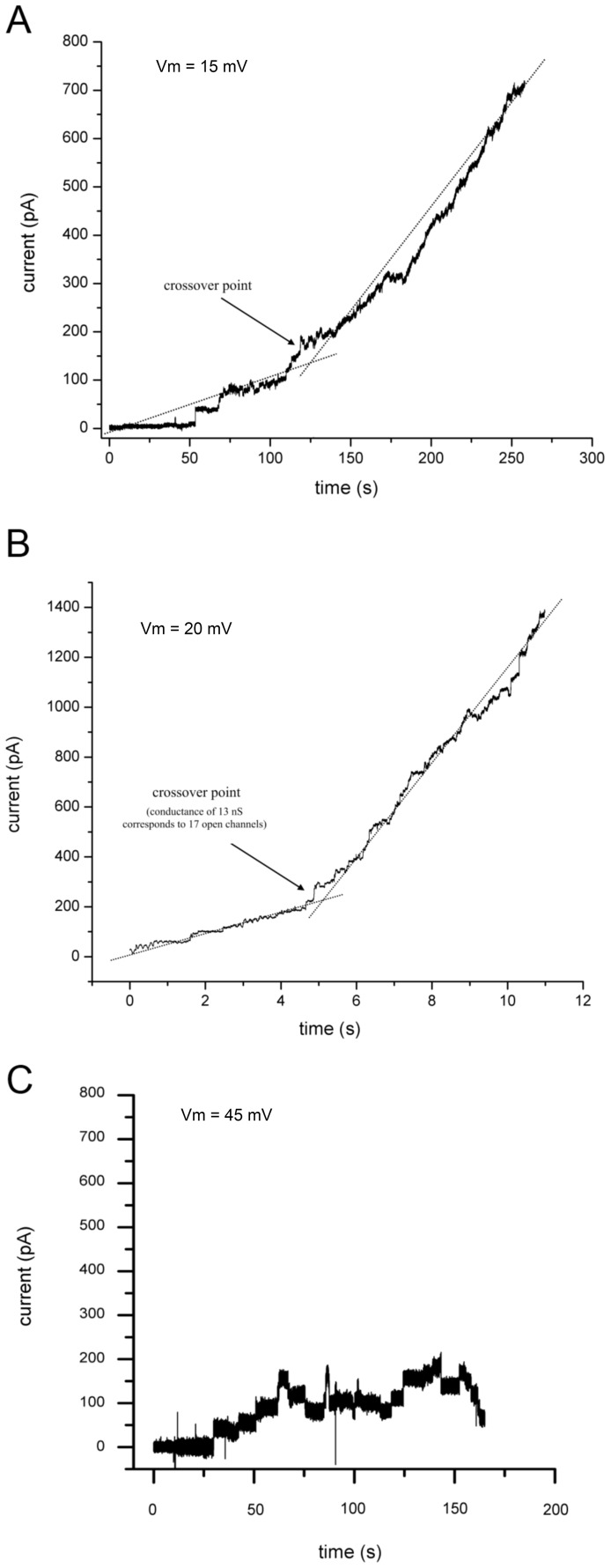
Aggregate behavior of Cx43 and Cx43eGFP hemichannels. As the number of open (A) Cx43 channels and open (B) Cx43eGFP channels reach a critical value, the change in conductance increases rapidly. (C) When ten-fold fewer Cx43eGFP proteoliposomes are added to the chamber, a change in the rate of conductance increase does not occur.

It has been proposed that aggregation or clustering is necessary for hemichannel opening [23]. Similarly, we observed a significant change in the rate of discrete conductance jumps when the open pore number was above a critical value of about 17 hemichannels (Figure 3B). By dividing the total conductance at the crossover point by the average conductance per hemichannel, we obtain a critical open pore number of 17±1.3 (n = 9). If we assume that only 10% of the channels in the bilayer plaque are actually open [23], our predicted transition number of 170 correlates reasonably, but slightly lower than *in vivo* values of 200–400 hemichannels reported for coupled plaque behavior [23]. This change appeared to be independent of time (Figure 3A and 3B) and bilayer thickness (see Text S1 and Figure S1E and S1F), as monitored via capacitance, suggesting changes in the lipid environment were not a significant factor. Furthermore, diluting the proteoliposomes with mock vesicles did not change the crossover value. When ten-fold fewer Cx43eGFP vesicles were added to the chamber, stepwise insertion was seen representing open hemichannel insertion; however, the crossover to rapid conductance increase never occurred, with the collective open channel conductance remaining stable and below the critical number (Figure 3C).

**Figure 4:**
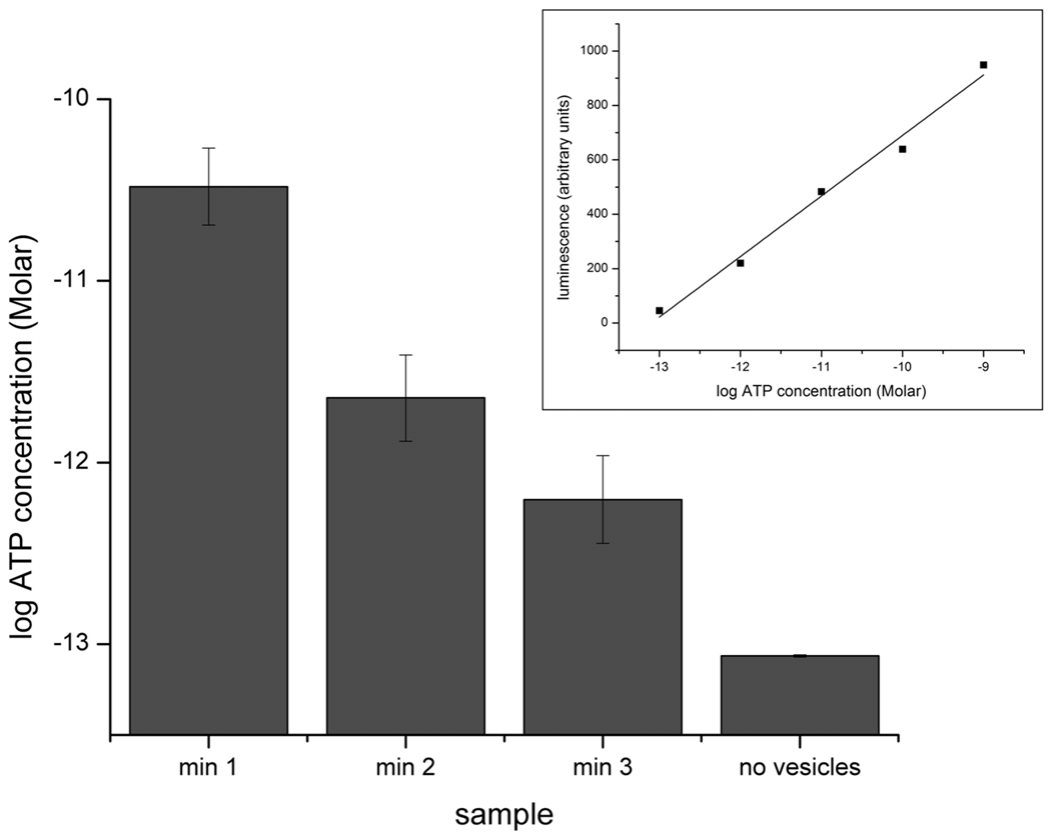
Vesicle fusion rate decreases with time. Vesicles loaded with ATP are added to the chamber containing an intact lipid bilayer. As the vesicles fuse to the bilayer, their contents are delivered to the bottom chamber. The concentration of ATP in the bottom chamber was sampled 1, 2, and 3 minutes after the addition of the vesicles (n = 3). ATP without vesicles was added to ensure the ATP delivery to the bottom chamber was dependent on vesicle fusion. The inset is a standard curve generated using known amounts of ATP.

To precisely examine vesicle fusion over time, protein-free vesicles were loaded with adenosine triphosphate (ATP) and added to the top chamber. As these vesicles fused, ATP was delivered to the bottom chamber. A custom perfusion system extracted liquid from the bottom chamber every minute and a luciferase bioluminescence assay was used to determine the concentration of ATP in the bottom chamber during the first several minutes after vesicle addition (Figure 4). As a control, free ATP was added to the top chamber showing no passage through the artificial bilayer. We observed that most of the vesicles fused and delivered ATP to the bottom chamber within the first minute after injection. Minutes two and three continued the exponential decrease with each containing ten-fold less ATP than the minute before. The observation that 92% of all vesicle fusion occurs within the first minute suggests that the accelerated conductance changes seen several minutes later cannot be adequately explained by new connexon delivery to the membrane. Instead, the accelerated conductance transition suggests that once enough hemichannels aggregate in the bilayer many of the silently inserted hemichannels open due to local interactions with other connexons; crossing over from single open hemichannels to collective clustering behavior needed for plaque formation.

Nevertheless, even in diluted form the number of connexons in 1 uL of vesicles (100 ng Cx43) is very large. While many of the proteins may remain unincorporated, we expect at least a fraction to be silently fused to the artificial bilayer. We can estimate the number of hemichannels inserted into the bilayer from the concentration of ATP delivered to the bottom chamber using the extruded vesicle size and concentration of ATP within the liposomes. After one minute, the ATP concentration in the bottom chamber was 3.3×10^−11^ M, which is equivalent to about 1×10^9^ vesicles fusing to the bilayer assuming 100 nm vesicles containing 500 mM ATP. Although this only represents about 0.2% of the total number of vesicles added to the top chamber, it still eclipses the total number of open hemichannels observed in the bilayer by several orders of magnitude, suggesting that there is a significant number of hemichannels incorporated into the bilayer that are not open. These silently incorporated proteins could provide the fuel to a growing plaque explaining the accelerated conductance increase. While we were unable to explore hemichannel interactions optically, the number of hemichannels involved, the diffusion constant for Cx43 in the bilayer [24], and the restricted surface area of the membrane, would produce a mutltitude of connexon-connexon interactions within a timescale of a few minutes. Furthermore, clustering of these hemichannels increases at lower pH [24]. To further investigate the possibility of silent incorporation, we examined the effects of dephosphorylation on a stable non-conducting membrane after vesicles had been added without observable channel insertion.

### Hemichannel Openings Due to Dephosphorylation

**Figure 5:**
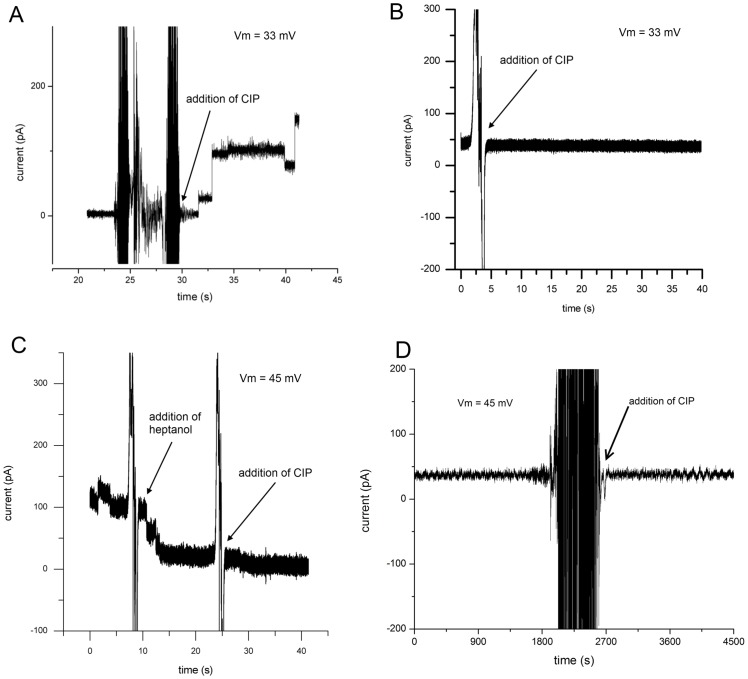
Hemichannel openings due to dephosphorylation. (A) After adding 10 units of CIP to the chamber, Cx43 hemichannel mediated increases in conductance were observed. (B) CIP did not cause increases in conductance when Cx43 proteoliposomes that were previously dephosphorylated using CIP, were fused to the membrane. (C) CIP did not cause increases in conductance after Cx43 conductance was blocked by adding heptanol to the chamber at a final concentration of 10 µM. (D) Adding CIP caused Cx43 hemichannel mediated increases in conductance and the addition of heptanol to the top and then bottom chamber blocked the conductance.

It has been shown that phosphorylating Ser-368 of Cx43 with protein kinase C (PKC) creates a conformational change in the protein which decreases its permeability to organic solutes [14]. In the cellular environment, phosphorylation plays a major role in the lifecycle of connexin as well as gating single hemichannels not incorporated into gap junctions [25]. Further, calf intestinal phosphatase (CIP) has been proven to dephosphorylate Ser- 368 on Cx43, causing the channel to remain open and permeable to organic solutes [14]. Here we show that dephosphorylation by CIP also increases the ionic conductance of Cx43 hemichannels in a sharp, stepwise manner. Isolation of the effect of CIP on the conductance of Cx43 through interaction with Ser-368 was confirmed via the generation of a Cx43 Ser-368 mutant. The Ser-368 mutated form of Cx43 was found to have a single-channel conductance of 765±30 pS (*n* = 30) and failed to produce an electrical response to CIP application (Figure 5). Since Sf9 cells are capable of Cx43 phosphorylation [14], a fraction of the inserted hemichannels were assumed to be closed as a result of this process. After adding proteoliposomes to the artificial bilayer with no observed conductance changes, the chamber was perfused to remove unbound vesicles and CIP was added to both top and bottom chambers. Directly after the addition of CIP, Cx43 hemichannel mediated conductance increases were observed (Figure 5A) in 5 out of 6 experiments. The average time from addition of CIP to hemichannel opening was 5.8±2.1 seconds (n = 5). Unlike Cx43, attempting to dephosphorylate Cx43eGFP while in the bilayer was largely unsuccessful. This could mean that the addition of eGFP to the c-terminus hinders access to the phosphorylation site at Ser-368. Rephosphorylating Cx43 with PKC in the BLM was not possible since the enzyme buffer requirements were incompatible with our acidic experimental conditions. So to isolate the effect of CIP on the conductance of Cx43 a S368A mutant was made through point mutation of the Cx43 plasmid. The S368A form of Cx43 was found to have a single-channel conductance of 765±30 pS (n = 30) and failed to permit a current signal upon application of CIP (Figure 5). This result confirmed the purified S368A Cx43 protein was insensitive to CIP.

Our in-vitro, real-time dephosphorylation with CIP shows that the process is fast, and suggests that the phosphorylation site at Ser-368 is accessible while Cx43 is incorporated into the membrane. To ensure that the CIP did not have a nonspecific effect on the BLM experimental setup, Cx43 proteoliposomes were treated with CIP prior to their use in any BLM experiments. Because the Cx43 in these liposomes were already dephosphorylated, added CIP then had no effect on conductance (Figure 5B). To further demonstrate that the CIP-mediated increases in conductance were specific to Cx43, after CIP addition the proteins were blocked with the addition of Heptanol (Figure 5D). Similarly, open Cx43 hemichannels were blocked with heptanol prior to CIP addition and did not show any sensitivity to the enzyme afterward (Figure 5C). Heptanol was selected since it has been shown to disrupt gap junctions *in-vivo* [26]. In addition to heptanol, several other known connexin blockers were also explored for their ability to abolish hemichannel conductance.

### Connexin Blockers

There has been some debate over the exact role of certain gap junction blockers in disrupting, closing or preventing normal intercellular communication. Although commonly referred to as ‘blockers’, some of the molecules proven to inhibit intercellular communication actually work indirectly through disruption of gap junction trafficking and assembly. Also, gap junction blockers may affect ionic conductance differently than the transfer of small organic solutes, simply by differences in relative pore occlusion. Most blockers are evaluated based on their ability to inhibit the transfer of Lucifer Yellow or a similar compound. Here we examine traditional gap junction blockers that have been shown to reduce the transfer of organic solutes in previous studies under physiological conditions [26]–[34]. Using our system, the blocking effect is specific to ionic conductance and individual purified proteins.

**Figure 6:**
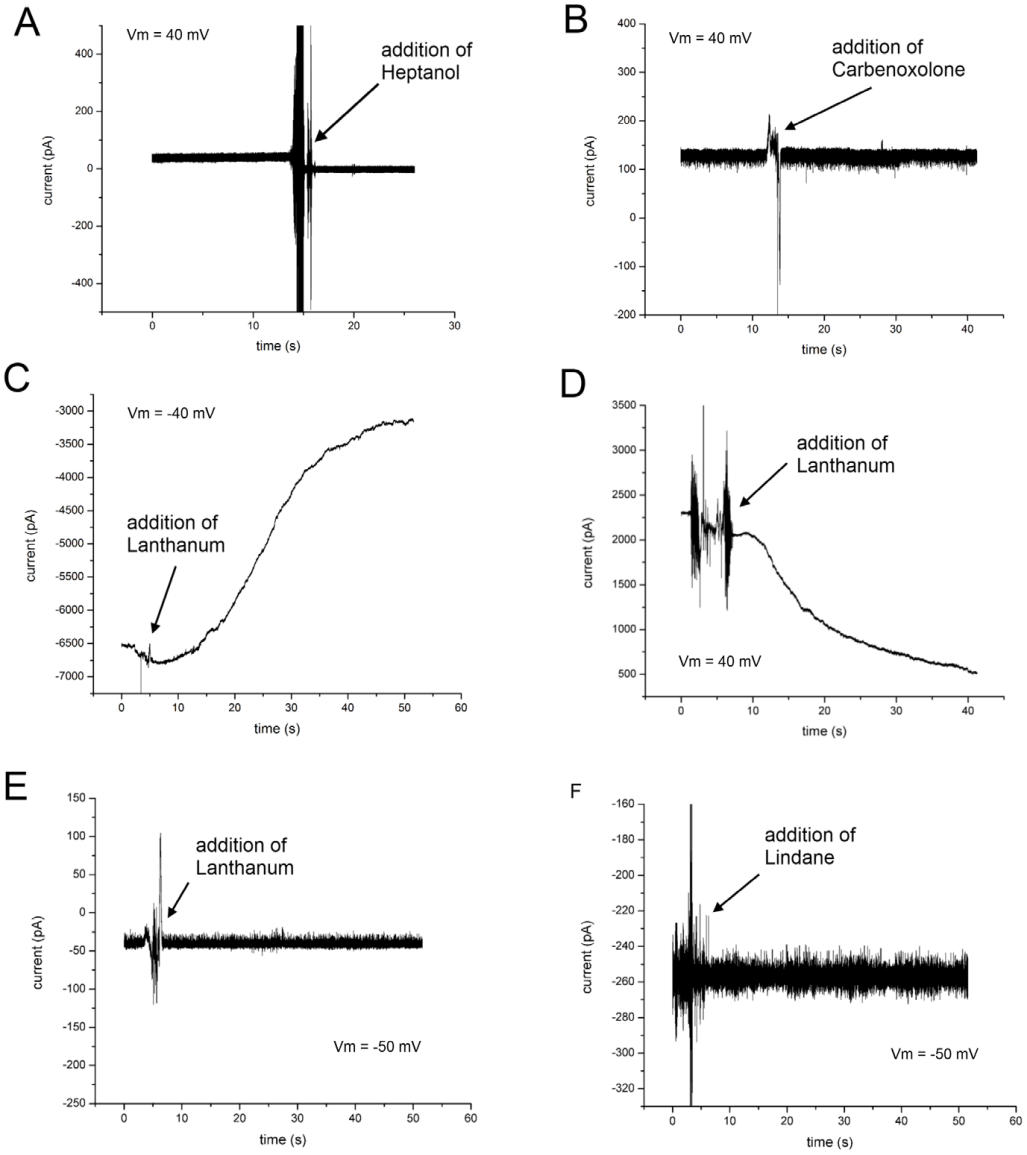
Hemichannel blockers in an artificial bilayer environment. (A) The addition of Heptanol to conducting Cx43 hemichannels terminated ionic current. (B) The addition of Carbenoxolone had no effect on open Cx43 hemichannels. (C) Lanthanum blocked current flowing through a collective aggregate of Cx43 hemichannels. (D) Lanthanum also blocked current flowing through a collective aggregate of Cx43eGFP hemichannels. (E) Lanthanum did not decrease current flowing through a single Cx43 hemichannel. (F) Lindane had no effect on the conductance of Cx43 hemichannels when added to the chamber.

Heptanol for instance, has been found to block gap junctions in ionic and dye transfer studies [26], [27]. When added to either side of the chamber at a final concentration of 10 µM, Heptanol caused a stepwise decrease of current (Figure 6A). Conversely, another common blocker, Carbenoxolone [26], [28], [29] did not alter hemichannel conductance (Figure 6B) in our experiments. Although this blocker has been proven to disrupt hemichannel packing and aggregation *in vivo* [28], this result lends support to the theory that Carbenoxolone acts indirectly by intercalating into the cell membrane, altering the local lipid environment to hinder plaque formation [26], [29].

The role of Lanthanum, a blocker from the family of trivalent Lanthanides has remained controversial. It has been proposed to physically block the pore in spite of pore lumen-ion size differences, as well as gate the pore indirectly through a secondary cellular messenger [17], [23], [30]. *In vitro* testing of Lanthanum revealed a marked decrease in conductance for both Cx43 (Figure 6C) and Cx43eGFP (Figure 6D) immediately after addition to either side of the chamber after several connexons had inserted. However, Lanthanum did not appear to block ionic current through single connexon hemichannels (Figure 6E). The minimum amount of trivalent cation required for observable blockage of collective hemichannel insertions was 43.3±3.3 µM (n = 3). Lanthanum caused decreased conductance only after large collections of hemichannels had amassed. Switching the voltage polarity in an attempt to force the Lanthanum cations in the reverse direction did not result in a reversal of the blockage of current. While not excluding ion protein interactions, these results contradict physically occluding the pore, and suggest that Lanthanum may act on Cx43 in an exogenous manner such as interacting with the c-terminus, or altering the lipid environment, the latter being a recognized property of lanthanides [31]. Although this does not elucidate the exact method by which Lanthanum blocks Cx43 channels, it does distinguish that Lanthanum can reduce gap junction conductance without any secondary cellular messengers or signaling pathways, mirroring recent results with Cx43-eGFP in planar bilayers [13].

Lindane, a former widely used agricultural pesticide, was another gap junction blocker examined under BLM. This organochlorine has been reported to rapidly inhibit gap junction communication and under chronic exposure, produce a loss of phosphorylated Cx43 and gap junction formation [32], [33]. Lindane is also known to be a neurotoxin and a non-genomic carcinogen, building up in the ovaries and testes [34]–[36]. Like Carbenoxolone, the addition of Lindane to open Cx43 hemichannels appeared to have no effect on conductance (Figure 6F), indicating a more indirect inhibitory role, especially when considering the multitude of deleterious effects on the nervous, circulatory and endocrine systems.

## Discussion

Cx43, Cx43eGFP and Cx43-Ser368 hemichannels were inserted into an artificial bilayer. Conductances of single channels were measured and significant changes in the rate of current increase were observed when the number of open hemichannels surpassed approximately 17. When considering the amount of silently incorporated Cx43, the observation that the critical numbers of hemichannels required for both opposed and unopposed plaque formation is similar suggests that initial clustering may begin as an independent cellular process. Cellular adhesion machinery facilitates clustering at cell to cell contact points by reducing the surface area available for lateral diffusion [37], making unopposed plaque formation unlikely. However, our results indicate that clustering might be independent of cell machinery and as a result, proteins that assist plaque formation *in vivo* such as ZO-1 augment this process, rather than being explicitly required. Indeed, it has been shown that fusion of GFP to the Cx43 carboxyl terminus in HeLa cells causes a loss of interaction with ZO-1 and uncontrollable growth of plaque size [38]. In our experiments, connexon lateral diffusion is limited only by the aperture size, making any additions to a plaque periphery unregulated.

Contrary to previous cellular studies [9], [15] we observed individual Cx43eGFP channels with sub-conductive states of approximately 35%. The conductance value and gating timescales are consistent with in-vivo results for Cx43, but were not observed for every Cx43eGFP which inserted. This gating mechanism is still not fully understood, but if these events were regulated by protein-protein interactions, such as inter connexon c-terminus interactions then unpaired hemichannels may be free to gate in this manner. EGFP is known to dimerize [13], albeit weakly, and such pairing could limit subunit mobility if complexed with other connexons.

CIP was used to dephosphorylate Cx43 hemichannels while in the bilayer, resulting in a stepwise increase of ionic conductance consistent with individual connexon openings. Gap junction blockers are commonly evaluated by their ability to inhibit organic solute transport or dye uptake. In this study we examined their effects on hemichannel ionic conductance using an in-vitro artificial lipid bilayer. Heptanol reduced the conductance of both single and large groups of hemichannels in a stepwise manner. Lanthanum also decreased ionic current, but only for collectively conducting hemichannels and had no measurable effect on individual channels. Lindane and carbenoxolone also had no effect on the conductance of hemichannels, in either large or small numbers.

One drawback to testing in a BLM environment is the necessity for highly conductive salt solutions. Lesser conductive salt solutions produce a lower signal to noise ratio, which is tolerable but not convenient. The other major limitation to our system is the deviation from resting cellular pH. It is not clear how an increase in protonation improves open hemichannel insertion, particularly in light of the *in vivo* data for acidification induced uncoupling of cells expressing Cx43 [18], but it appears that gap junction and hemichannel pH response may differ. This difference may be explained by the static, balanced pH in the BLM chamber versus the dynamic and asymmetric *in vivo* conditions, and the complex relationship between ammonium sulfate, connexin isoform and pH. Still, it remains valuable to evaluate the interaction of Cx43 with potential blockers at a slightly acidic pH as a model for their use during clinically relevant scenarios like myocardial infarction. Intracellular pH regulates excitability, contractility, and gap junction activity in cardiac muscle [39] and the intracellular pH of rabbit hearts has been shown to drop to below 6.0 during ischemia [40]. As a result, pH modulation of intercellular communication has been proposed as a means to limit the propagation of metabolic stress from damaged cells to unaffected surrounding tissue [41]. Because our blocker experiments were carried out in an acidic and highly conductive salt environment, these results do not completely rule out interactions that could take place in a healthy physiological environment. However, the Cx43-blocker interactions under these conditions remain clinically relevant to the pursuit of limiting cellular damage during comprehensive ischemia.

The benefits of testing the effects of various molecules on connexin independent of the cellular milieu may prove to outweigh these weaknesses. Although blocking and dye permeability assays are routinely used to identify connexins, these properties are not unique to gap junctions and are often exhibited by other ion channels. One example of this “cross-inhibition” is 5-Nitro-2-(3-phenylpropylamino) benzoic acid (NPPB), which has been found to inhibit connexin hemichannels and volume regulated anion channels at similar concentrations *in vivo* [26]. Using our approach, chemical libraries could be screened for compounds that specifically block or interact with Cx43. This would allow for identification of connexin specific results in an *in vitro* environment. Although Cx43 is the most widely studied gap junction, most of these studies are carried out *in vivo* and do not address the emergent functional differences between single hemichannels and plaques. Recently, compounds have been shown to affect hemichannels and gap junctions differently although in the same cell line and composed of the same type of connexin [42]. This highlights our system’s advantage of helping to elucidate the differences in plaque and individual hemichannel behavior. Manipulation of the gap junction plaque lifecycle has been proposed as a viable therapeutic target [41], [42]. Applying our *in vitro* system to test Cx43 independent of the cellular environment, on both hemichannel and gap junction behaviors, would be invaluable to this pursuit.

## Supporting Information

Figure S1 **Gel electrophoresis of purified connexin and controls.** (A) Purified Connexin43-eGFP protein is shown analyzed SDS-PAGE and (B) Purified connexin43 shown by western blotting using anti-Cx43 antibody. (C) A mock purification of Cx43 was carried out by harvesting Sf9 cells devoid of the pIEX4-Cx43 plasmid. The output was then used in the proteoliposome preparation and BLM fusion procedures. When added to the chamber containing a lipid bilayer, the vesicles prepared from the mock purification produced no changes in conductance. (D) When the buffer used in the chamber was added to conducting hemichannels, the conductance did not change. (E) Current recordings resulting from the application of a sawtooth voltage waveform before and (F) after the addition of proteoliposomes. These current values were used to calculate the thickness of the membrane to insure a bilayer was formed and that its thickness did not change during vesicle fusion.
(TIF)

Table S1 **Comparison of single hemichannel conductance values for Cx43 and Cx43eGFP.** *A ratio of 3.607 was calculated by measuring the conductance of our KCl buffer (500 mM KCl, 2.5 mM MES, pH 5.7) and the NaCl chamber buffer (140 mM NaCl, 5.4 mM KCl, 1 mM MgCl2, 10 mM HEPES, pH 7.4) prepared as described [2]. Conductances are mean ± s.d.
(DOC)

Text S1 Bilayer thickness calculation.
(DOC)
