# Supplementary figures and images for: Electrophysiology of Single and Aggregate Cx43 Hemichannels
Source: PLoS One. 2012 Oct 24;7(10):e47775. doi: 10.1371/journal.pone.0047775 (PMC3480394; doi:10.1371/journal.pone.0047775)

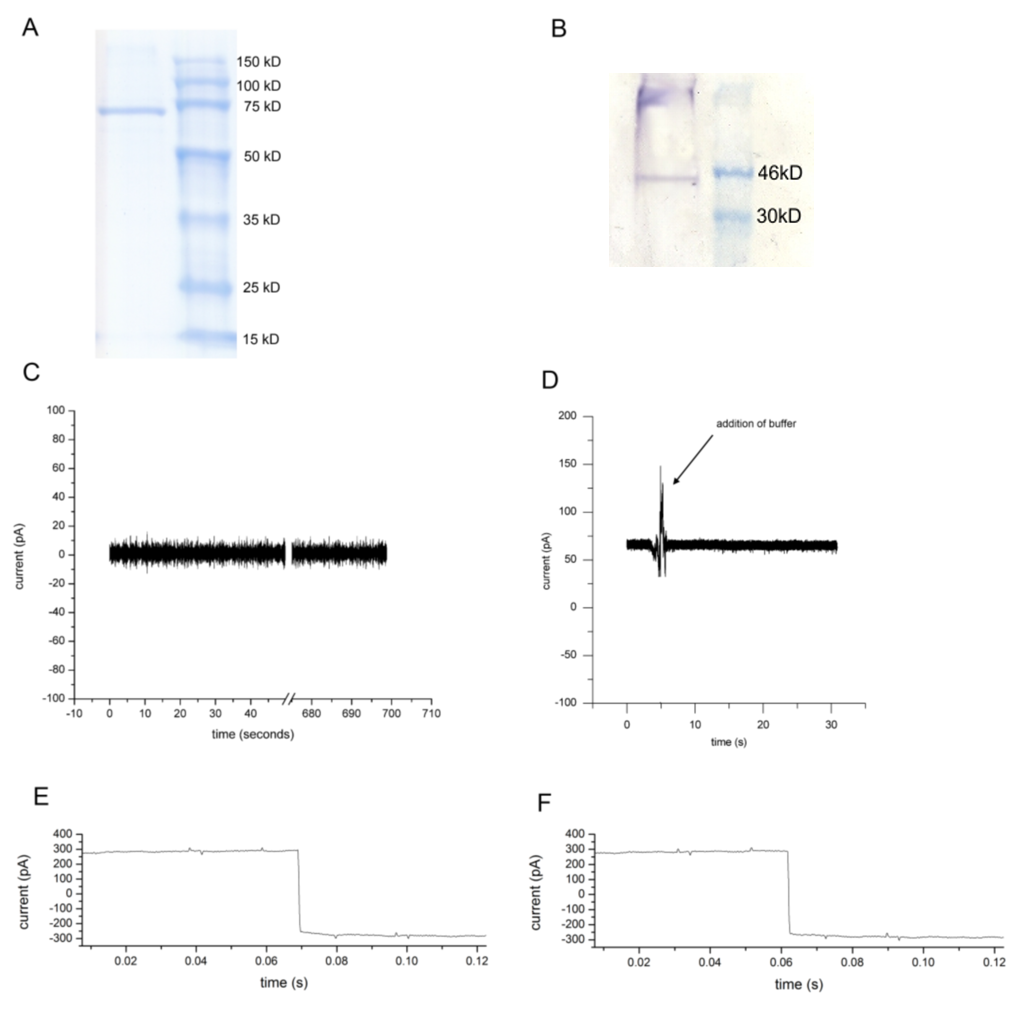

Supplement: Figure S1 — Gel electrophoresis of purified connexin and controls. (A) Purified Connexin43-eGFP protein is shown analyzed SDS-PAGE and (B) Purified connexin43 shown by western blotting using anti-Cx43 antibody. (C) A mock purification of Cx43 was carried out by harvesting Sf9 cells devoid of the pIEX4-Cx43 plasmid. The output was then used in the proteoliposome preparation and BLM fusion procedures. When added to the chamber containing a lipid bilayer, the vesicles prepared from the mock purification produced no changes in conductance. (D) When the buffer used in the chamber was added to conducting hemichannels, the conductance did not change. (E) Current recordings resulting from the application of a sawtooth voltage waveform before and (F) after the addition of proteoliposomes. These current values were used to calculate the thickness of the membrane to insure a bilayer was formed and that its thickness did not change during vesicle fusion. (TIF) [file pone.0047775.s001.tif]
