# Supplementary material for: Electrophysiology of Single and Aggregate Cx43 Hemichannels
Source: PLoS One. 2012 Oct 24;7(10):e47775. doi: 10.1371/journal.pone.0047775 (PMC3480394; doi:10.1371/journal.pone.0047775)
Supplement: Table S1 — Comparison of single hemichannel conductance values for Cx43 and Cx43eGFP. *A ratio of 3.607 was calculated by measuring the conductance of our KCl buffer (500 mM KCl, 2.5 mM MES, pH 5.7) and the NaCl chamber buffer (140 mM NaCl, 5.4 mM KCl, 1 mM MgCl2, 10 mM HEPES, pH 7.4) prepared as described [2]. Conductances are mean ± s.d. (DOC) [file pone.0047775.s002.doc]

|  | Results | Contreras, et al [15] |
| --- | --- | --- |
| Cx43 | 753 ± 31 pS | 794 ± 36 pS* |
| Cx43eGFP | 783 ± 53 pS | 804 ± 32 pS* |
