# Supplementary material for: Electrophysiology of Single and Aggregate Cx43 Hemichannels
Source: PLoS One. 2012 Oct 24;7(10):e47775. doi: 10.1371/journal.pone.0047775 (PMC3480394; doi:10.1371/journal.pone.0047775)
Supplement: Text S1 — Bilayer thickness calculation. (DOC) [file pone.0047775.s003.doc]

**Bilayer Thickness Calculation**

The bilayer in this system is typically modeled as a simple parallel plate capacitor where the “plates” are represented by the ionic charge on either side of the lipid membrane. The relevant equation is:

$C=\frac{\varepsilon_{0}\varepsilon A}{d}$ (1)

Where C is capacitance in farads (F), ε_0_ is the permittivity of free space (8.85x10^-12^ (F/m)), ε is the dielectric constant of the lipid membrane (2), A is the surface area (m^2^), and d is the membrane thickness (m). Here, we will assume the simplest form of this model: a system consisting of two dielectric constants, ε = 2 for the lipid core, and 80 for the surrounding aqueous environment. Using this we can calculate expected membrane thickness, assuming an exact aperture diameter or vice versa, using the measured membrane capacitance. Membrane capacitance is a measured quantity derived from the simple relationship between current and charge:

$C\frac{dV}{dt}=I$ (2)

At a current of 280 pA (Figure S1E and S1F) during the application of 25 mV voltage ramp lasting 63 ms, *C* is equal to 710 pF. Using a partition diameter of 500 µm and the permittivity and dielectric constants mentioned previously, the lipid membrane is calculated to be 4.9 nm thick. This is within the reported thickness of a bilayer formed with DPhPC [1].

**References**

1. Wendell D, Jing P, Geng J, Subramaniam V, Lee TJ, et al. (2009) Translocation of double-stranded DNA through membrane-adapted phi29 motor protein nanopores. Nature Nanotech 4: 765-772.
